# Telomeres are elongated in rats exposed to moderate altitude

**DOI:** 10.1186/1880-6805-33-19

**Published:** 2014-07-04

**Authors:** Yaping Wang, Wen-di Zhou, Yingzhong Yang, Lan Ma, Yanxia Zhao, Zhenzhong Bai, Ri-Li Ge

**Affiliations:** 1 Research Center for High Altitude Medical Sciences, University School of Medicine, 810001 Xining, Qinghai, China; 2 Department of Pediatrics, Huai'an First People’s Hospital, Nanjing Medical University, 6 Beijing Road West, 223300 Huai'an, China; 3 Internal Medicine, Research Center for High Altitude Medical Sciences, Qinghai University School of Medicine, 16 Kunlun Rd, 810001 Xining, Qinghai, China

**Keywords:** Telomere, Altitudes, Hypoxia-inducible factor 1α, Superoxide dismutase, Malondialdehyde

## Abstract

**Background:**

Leukocyte telomere length has been shown to be associated with life span. Hypoxia-associated changes of telomere length have been detected in cell cultures, but no *in vivo* studies have reported the changes of telomere length under different hypoxic conditions. This study aimed to evaluate the effects of altitude on telomere length in rat leukocytes.

**Methods:**

One hundred and ten male Wistar rats were randomized into 3 groups and maintained at sea-level (altitude of 10 m) (SL group, n = 10), moderate altitude (2,260 m) (MA group, n = 50), or simulated high altitude (5,000 m (SHA group, n = 50). The last two groups were further divided into 5 subgroups and exposed to hypoxia for 1, 3, 7, 15, or 30 days (n = 10). The leukocyte telomere length, hemoglobin concentration, red blood cell count, hematocrit, and plasma levels of telomerase reverse transcriptase (TERT), hypoxia-inducible factor 1α (HIF-1α), malondialdehyde (MDA) and superoxide dismutase (SOD) were measured.

**Results:**

Leukocyte telomere length was significantly longer in the MA group than in the SL or SHA groups, and the TERT expression changed in a similar manner as the leukocyte telomere length. However, HIF-1α level was significantly higher in both MA and SHA groups than the SL group. SOD level was decreased and MDA level was elevated in SHA group.

**Conclusions:**

The telomere length of blood leukocytes is elongated at a moderate altitude, but not at a high altitude. A mild hypoxic state may increase telomere length.

## Background

Telomeres are specialized nucleoprotein structures at the ends of chromosomes that are composed of thousands of TTAGGG/AATCCC repeats and associated proteins [1]. A critical function of telomeres is to cap and protect the ends of chromosomes from degradation and end-to-end fusions, which otherwise would result in the loss of genetic information and chromosomal instability [2]. It has been well established that excessive shortening of the telomeres can lead to genomic instability or activation of cellular damage responses, such as cellular senescence and apoptosis [3]. Telomeres are also essential for cell survival and replicative capacity of dividing somatic cells.

Telomere shortening occurs as a consequence of the proliferation along with each cell division. Increasing evidence indicates the association between the significant telomere shortening of hepatocytes and the replicative senescence of a non-dividing state [4]. Leukocyte telomere length was shown to be positively related to life span, thereby making it a potential indicator of longevity [5]. Recent studies have shown that a number of factors have effects on the telomere length in somatic cells, including low temperature, ultraviolet radiation, smoking, diet, mental stress, inflammation and oxidative stress [6-11]. A topic of increasing importance in recent years concerns the role of hypoxia. Telomere length has been shown to vary considerably among different hypoxia levels and cell types [12-14]. However, most of these studies are *in vitro*, and few studies have investigated the effects of hypoxia on telomere length *in vivo*.

In this study, we measured the telomere lengths of peripheral blood leukocytes in the rats exposed to different altitudes (10 m, 2,260 m, 5,000 m) for different periods (1, 3, 7, 15, 30 days), with the aim to elucidate the effect of altitude and hypoxia on aging.

## Methods

### Experimental animals

All animal experiments were approved by the Ethical Committee of Qinghai University. A total of 110 male Wistar rats (approximately 150 to 180 g) were used in this study. All the rats were maintained in a controlled environment at 18 to 20°C with unlimited access to food and water on a 12-hour light/dark cycle, and then randomized into one of the following three groups. In the sea-level (SL) group, ten rats were maintained in Nanjing where the altitude was about ten meters. In the moderate altitude (MA) group, 50 rats were transported from Nanjing to Xining where the altitude was about 2,260 m, and further divided randomly into the 1-, 3-, 7-, 15-, 30-day subgroups (MA 1, 3, 7, 15, 30), with 10 rats in each subgroup. In the simulated high altitude (SHA) group, 50 rats were put into a hypobaric chamber in Xining where an altitude of 5,000 m was simulated, and further divided randomly into the 1-, 3-, 7-, 15-, 30-day subgroups (SHA 1, 3, 7, 15, 30), with 10 rats in each subgroup.

### Measurement of hemoglobin (HGB), red blood cell count (RBC) and hematocrit (HCT)

Venous blood was taken from the rats and HGB, RBC and HCT were measured using an automatic blood cell analyzer (BC-2300, Mairui Biotec, Wuhan, China) following the manufacturer's instruction.

### DNA isolation and quantitative PCR

Genomic DNA was isolated from peripheral blood leukocytes using a Blood DNA extraction kit (Tiangen Biotech, Beijing, China) following the manufacturer’s instruction, and dissolved in TE buffer (pH 8.0) at the concentration of 25 ng/μl. Quantitative PCR was performed using the SYBR® Select Master Mix (Takara, Dalian, China) with an ABI PRISM 7500 (Applied Biosystems, Foster City, CA, USA). The parameters were as follows: 95°C for 30 seconds, followed by 40 cycles of 95°C for 5 seconds and 60°C for 34 seconds. Primers used were as follows: Tel-1 5’GGT TTT TGA GGG TGA GGG TGA GGG TGA GGG TGA GGG t-3’, and Tel-2 5’-TCC CGA CTA TCC CTA TCC CTA TCC CTA TCC CTA TCC CTA-3’; AT1 rat-f 5’-ACG TGT TCT CAG CAT CGA CCG CTA CC-3’ and AT1 rat-r 5’-AGA ATG ATA AGG AAA GGG AAC AAG AAG CCC-3’ (Invitrogen, Carlsbad, CA, USA). The quantities of telomeric DNA were normalized to that of AT1. The relative telomere length was determined by calculating the relative ratio of telomere (T) and single gene copy AT 1 receptor (S), expressed as T/S ratio.

### Plasma levels of TERT, HIF-1α, SOD and MDA

The plasma samples were separated from the blood. The plasma levels of telomerase reverse transcriptase (TERT) and hypoxia-inducible factor 1α (HIF-1α) were detected using an ELISA kit (USCN Life Science Inc., Atlanta, GA, USA), and the plasma levels of superoxide dismutase (SOD) and malondialdehyde (MDA) were detected by WST-1 and TBA assay (SOD and MDA kit, Jiancheng Biotech Ltd., Nanjing, China) according to the manufacturer’s protocols.

### Statistical analysis

All data were presented as mean ± SD and analyzed using the SPSS version 12 statistical analysis package (SPSS Inc., Chicago, IL, USA). Statistical analysis was performed by one-way ANOVA, followed *post hoc* test. *P*-values less than 0.05 were considered statistically significant.

## Results

### Hematological changes in rats exposed to different altitude

**Figure 1:**
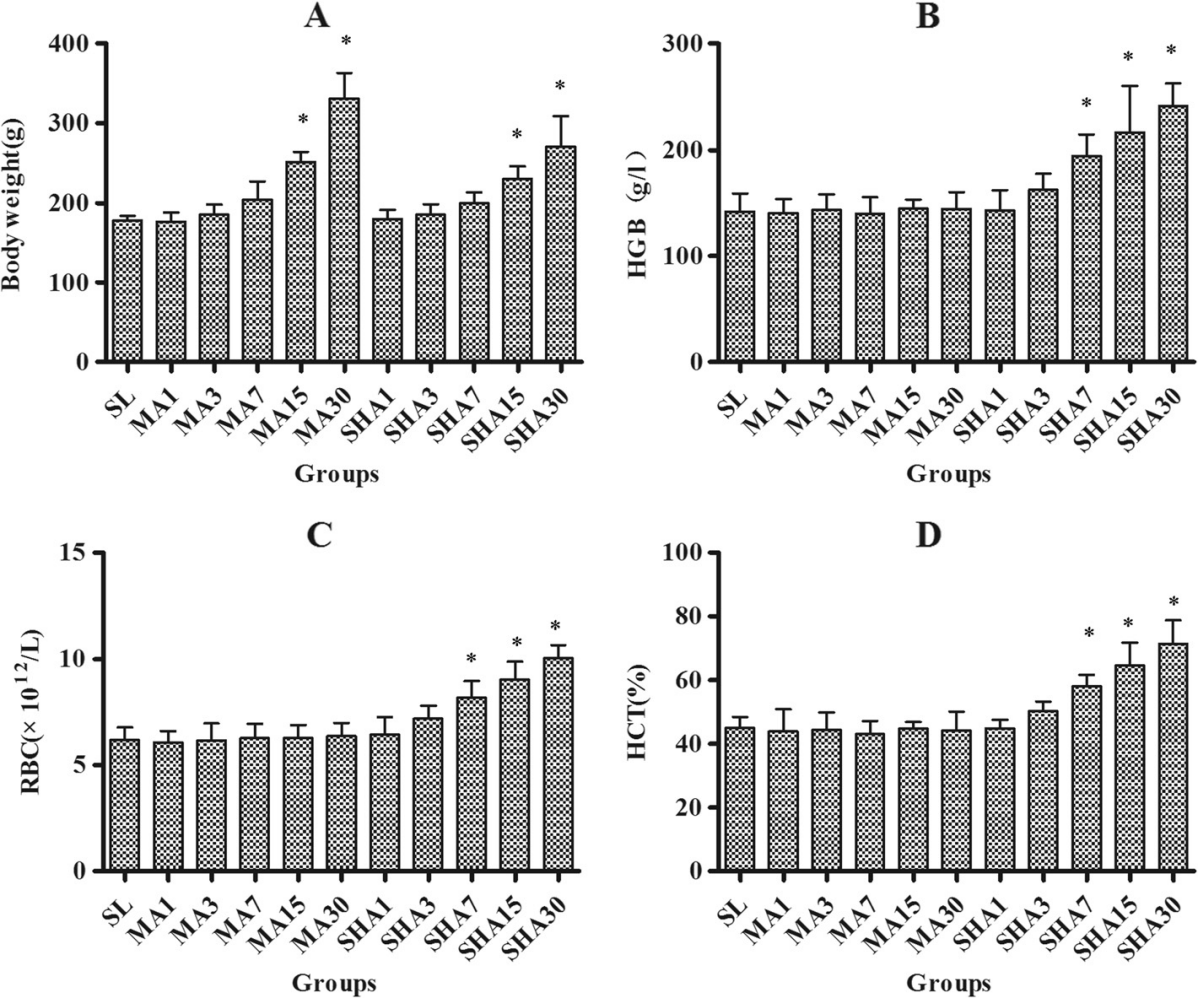
**Body weight (A), hemoglobin (HGB) (B), red blood cell count (RBC) (C) and hematocrit (HCT) (D) of rats exposed to different altitudes for 1, 3, 7, 15, and 30 days.** The data were shown as mean ± SD, n = 10. *P < 0.05 versus SL group.

We found that the rats in SHA group had reduced food intake and physical activity, and their body weights increased at a slower rate than those in the MA group (Figure 1A). No significant changes were observed in the HGB, RBC and HCT in rats in a moderate hypoxic environment. However, HGB, RBC and HCT were increased significantly over time in rats exposed to an extreme hypoxic environment (Figure 1B-D, *P* < 0.05). These data demonstrate that the hypoxic (at a simulated altitude of 5,000 m) rat model was established successfully.

### Telomere length of leukocytes from rats exposed to different altitude

**Figure 2:**
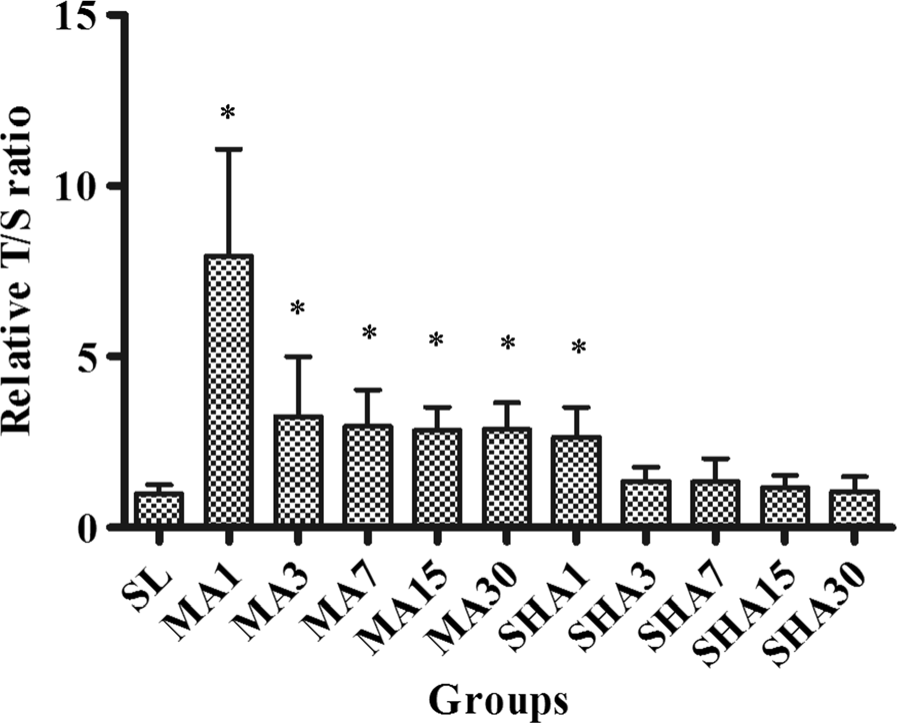
**Telomere length of leukocytes of rats exposed to different altitudes for 1, 3, 7, 15, and 30 days.** The telomere length was expressed as relative telomere to single gene copy AT 1 receptor (T/S) ratio. The data were shown as mean ± SD, n = 10. *P < 0.05 versus sea-level (SL) group.

The relative T/S ratios for each group are shown in Figure 2. It was evident that the telomeres were significantly longer in MA group than in the SL or SHA group (*P* < 0.05). However, there was no significant difference between the SL and SHA group. Another noteworthy feature was that in the MA or SHA group, telomere length was increased on the first day of exposure to hypoxic environment, but then shortened with further exposure. The main difference was that in the MA group, telomere length was significantly elongated on the first day, and then shortened to a level that was still longer than that in the SL group; whereas in the SHA group, the telomere length was slightly elongated on the first day, and then shortened to a level that was almost the same as that in the SL group. These results suggest that telomeres are elongated in a mildly, rather than a highly, hypoxic environment.

### Plasma levels of TERT, HIF-1α, SOD and MDA in rats exposed to different altitude

**Figure 3:**
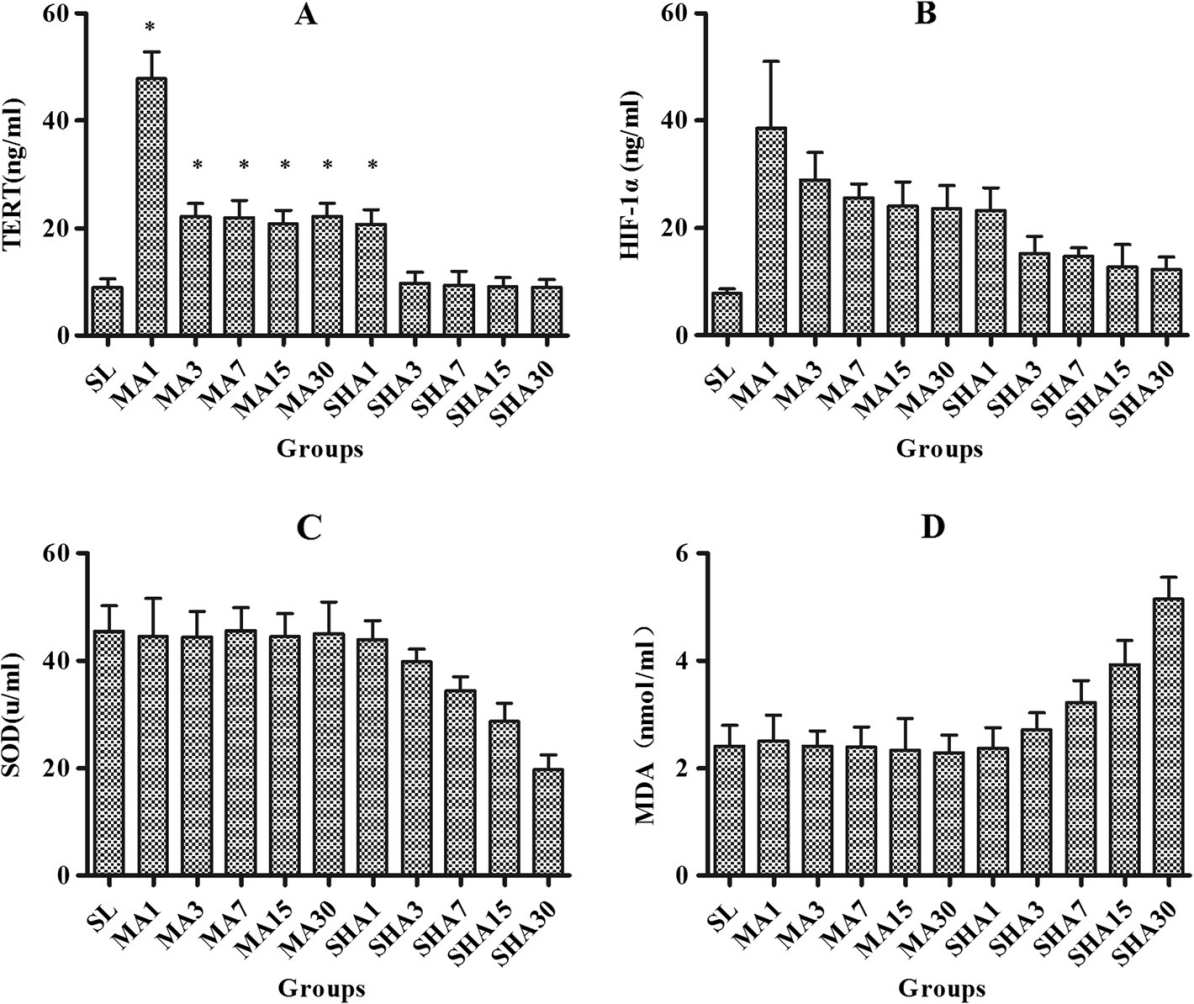
**Plasma levels of telomerase reverse transcriptase (TERT) (A), hypoxia-inducible factor 1α (HIF-1α) (B), superoxide dismutase (SOD) (C) and malondialdehyde (MDA) (D) in rats exposed to different altitudes for 1, 3, 7, 15, and 30 days.** The data were shown as mean ± SD, n = 10. *P < 0.05 versus sea-level (SL) group.

Next we detected the plasma TRET level and found that it changed in a similar manner to the leukocyte telomere lengths under the hypoxic conditions (Figure 3A). Compared to the SL group, TERT expression was significantly elevated in the MA group, but remained almost unchanged in the SHA group.Similarly, the plasma HIF-1α level changed in a similar manner to the leukocyte telomere length under hypoxic conditions (Figure 3B). HIF-1α expression was increased significantly in both MA and SHA groups compared to SL group. However, HIF-1α expression was much higher in the MA group than in the SHA group, suggesting that HIF-1α protein expression is more likely to be induced in a mildly hypoxic environment.SOD and MDA have been regarded as the indexes of the generation of reactive oxygen species (ROS). Therefore, we detected plasma SOD and MDA levels and the results showed that compared to the SL group, SOD and MDA levels were not significantly changed in the MA group, but SOD declined and MDA was elevated gradually in the SHA group (Figure 3C, D).

## Discussion

To our knowledge, this is the first *in vivo* study that compared leukocyte telomere length of rats exposed to different hypoxic conditions. Our results showed that the telomeres were significantly elongated in a mildly rather than highly hypoxic environment, and that the expression of TERT and HIF-1α changed in a similar manner as the leukocyte telomere length under the hypoxic conditions.

Telomere length is known to be maintained by telomerase, a specialized ribonucleoprotein complex consisting of a catalytic protein subunit TERT and a telomerase RNA component (TERC) that serves as a template for telomere extension during *de novo* addition of TTAGGG repeats onto chromosome ends [1]. TERT expression parallels telomerase activity in many multicellular organisms, indicating that telomerase activity can be determined indirectly by measuring TERT expression [15,16]. Exposure to hypoxia has been shown to activate telomerase due to its ability to increase TERT expression, which in turn results in telomere elongation [17,18]. The mechanisms responsible for hypoxia-induced TERT expression have been investigated, and it was shown that TERT expression was upregulated following the induction of HIF-1α during exposure to hypoxia and HIF-1α acted as an essential transactivator to induce TERT transcription [19-21].

Our results showed that the general condition of rats exposed to moderate hypoxia were good and without significant oxidative damage. However, the level of the antioxidant enzyme SOD declined and the oxide metabolite MDA gradually elevated in the SHA group, which suggests that antioxidative stress activity is decreased in the rats and renders them susceptible to oxidant injury. Previous studies have shown that severe hypoxia (low oxygen tension) can damage cellular components by producing ROS through the oxidation of DNA, proteins and lipids. Oxidative stress preferentially damages telomeric DNA regions and inhibits telomerase activity in *vitro* in various cell types, and has been suggested as a major cause of telomere shortening [22-25]. Therefore, it is possible that under severe hypoxic conditions oxidative stress-associated telomere DNA damage may counteract the increased telomerase activity and telomere elongation induced by HIF-1α.

In this study, HIF-1α expression was lower in the SHA group than in the MA group. It has been reported that increased production of ROS could downregulate HIF-1α, and impair the adaptive response of many oxygen-sensitive genes to hypoxia [26]. Interestingly, we found that the changes of HIF-1α and TERT expression and telomere length were correlated with the duration of exposure to hypoxia in both the MA and SHA groups. In general, HIF-1α and TERT expression and telomere length were increased on the first day of exposure to the hypoxic environment and then decreased rapidly to a stable level with further exposure. These results are consistent with previous findings that HIF-1α expression could be increased by acute hypoxia, but chronic exposure to hypoxia induced the downregulation of HIF-1α expression [27,28]. Notably, we observed that in the MA group telomere length was significantly elongated on the first day but was then markedly reduced afterwards, indicating that telomere length may be adapted after exposure to hypoxia.

## Conclusions

Our study showed that the telomeres of peripheral blood leukocytes were elongated at moderate altitude, but not at high altitude, and the telomere length was related to the periods of exposure to hypoxic environment. Furthermore, we found that HIF-1α and TERT expression and oxidative stress damage were parallel to the changes of telomere length. These findings support the beneficial effects of mild stress [29]. However, further studies are needed to explore alternative possibilities and elucidate the relationship between leukocyte telomere length and longevity in humans living at different altitudes.

## Abbreviations

SL: sea-level; MA: moderate altitude; SHA: simulated high altitude; TERT: telomerase reverse transcriptase; HIF-1α: hypoxia-inducible factor 1α; MDA: malondialdehyde; SOD: superoxide dismutase (SOD).

## Competing interests

The authors declare that they have no competing interests.

## Authors’ contributions

YW and WZ participated in the design and implementation of the study. YY, LM, YZ performed the measurements. ZB performed statistical analysis. RG superivsed the study. All authors read and approved the final manuscript.
